# Promoting Wellbeing and Resilience Amongst Resident Doctors

**DOI:** 10.1192/bjo.2025.10343

**Published:** 2025-06-20

**Authors:** Hemma Sungum, Laura Brunning, Chikwado Iwudibia, Madlen Griffiths

**Affiliations:** Cwm Taf Morgannwg University Health Board, Abercynon, United Kingdom

## Abstract

**Aims:** To develop and promote wellbeing amongst Resident doctors and embed this into the Core Trainee Committee (CTC).

**Methods:** This Quality improvement project was part of a response to Trainees’ wellbeing developed after noting the dissatisfaction of trainees with wellbeing in the 2024 GMC National training survey. It reported that over a fifth (21%) of trainees measured to be at high risk of burnout and over half (52%) described their work as emotionally exhausting to a very high or high degree.

Dr Sungum (wellbeing lead), devised the pathway using an internally generated traffic light system of the wellbeing department in the Trust and Deanery. Following this, some Core trainees trained as wellbeing activists to support their peers and created and distributed a wellbeing pathway and poster to that effect. We organised activities to improve wellbeing including monthly trainee socials and wellbeing lunch drop-ins. We created a Survey about wellbeing distributed amongst all Psychiatry trainees in the health board.

**Results:** 12 Psychiatry trainees were surveyed. 75% of respondents were aware of the wellbeing pathway we had created and found it useful and informative. 0% of respondents had used any resources from the wellbeing pathway. 83.3% of respondents found the trainee Socials beneficial to their wellbeing. They also gave feedback on how the wellbeing service can be improved for trainees. Below are some of their responses:

“When I started training there was no wellbeing talk. This initiative is fantastic. Keep up with the socials. Fundamentally, if the trainees felt more valued and cared for, wellbeing would certainly improve and reduce burnout.”

“For more trainees to know who to contact if struggling. But otherwise, good advertisement of resources. And socials have been a good way to meet more of my colleagues.”

“Having a dedicated time for informal discussion between colleagues as part of rotation transition – perhaps as part of the last Tuesday teaching session for 1 hour might be useful.”

**Conclusion:** Most Psychiatry trainees are aware of the wellbeing service but have not used it. Most respondents were keen on having more social activities as a way to improve their wellbeing. This Quality improvement project largely met its aim with room for further improvement.

Wellbeing is now embedded in governance as part of the CTC and is actively discussed with compassion in formal and non-formal settings.

